# Comparing the Long-Term Effectiveness of Sodium-Glucose Cotransporter 2 Inhibitors Versus Mineralocorticoid Receptor Antagonists as Add-On Therapies in the Management of Heart Failure: A Systematic Review

**DOI:** 10.7759/cureus.96616

**Published:** 2025-11-11

**Authors:** Varghese George, Mohit H Pillai, Vinni Jacob, Revanth MP, Anagha Vasant, Haroon Adnan

**Affiliations:** 1 General Medicine, East Lancashire Hospitals NHS Trust, Blackburn, GBR; 2 Nephrology, Kalyani Kidney Care Centre, Erode, IND; 3 Obstetrics and Gynaecology, Joseph Hospital, Chennai, IND; 4 Public Health Dentistry, Karpaga Vinayaga Institute of Dental Sciences, Chengalpet, IND; 5 Trauma and Orthopaedics, Ashford and St Peter's Hospitals, Chertsey, GBR; 6 General Surgery, East Lancashire Hospitals NHS Trust, Blackburn, GBR

**Keywords:** cardiovascular death, heart failure hospitalization, mineralocorticoid receptor antagonist, randomized controlled trial, sodium-glucose cotransporter 2 inhibitor

## Abstract

We sought to conduct a systematic review to compare the long-term effectiveness of sodium-glucose cotransporter 2 (SGLT2) inhibitors and mineralocorticoid receptor antagonists (MRAs) when they are added to guideline-mediated therapy to treat patients with heart failure. To undertake this task, we searched PubMed, MEDLINE, Scopus, and ClinicalTrials.gov from 2020 to 2025. Randomized controlled trials (RCTs) were selected if they investigated both of the pre-specified outcomes that measured long-term effectiveness. A total of five trials were included: four trials studying SGLT2 inhibitors and one trial studying MRAs. A total of 14340 heart failure patients were included from both cohorts: 8339 in the SGLT2 inhibitor cohort and 6001 in the MRA cohort. Both SGLT2 inhibitors and MRAs significantly reduced the risk of worsening heart failure events (SGLT2 inhibitors by 25.21% and MRAs by 17.93%) and cardiovascular deaths (SGLT2 inhibitors by 14.36% and MRAs by 7.16%) when compared to placebo. A head-to-head comparison of both drug classes showed that SGLT2 inhibitors fared better than MRAs in reducing worsening heart failure events and cardiovascular deaths.

## Introduction and background

Heart failure is a syndrome that limits the heart's capacity to adequately pump its contents, resulting in inefficient circulation of blood [1]. There are structural and functional causes that lead to this, such as ischemic heart disease, cardiomyopathies, valvular and rheumatic heart diseases, and hypertension [2]. Heart failure is a chronic disease that has debilitating effects, often presenting to the hospital as shortness of breath, fatigue, pulmonary oedema, and pedal oedema [3]. Heart failure has a prevalence of about 26 million people, with some estimates as high as 64 million affected worldwide, causing a strain on the healthcare infrastructure of various developed and developing countries [4,5]. Treating heart failure has become costlier; a recent study estimated the overall economic cost of treating heart failure at around $108 billion [6]. Treating heart failure requires a multidisciplinary approach, which involves lifestyle changes, cardiac rehabilitation, and medications like angiotensin-converting enzyme inhibitors, angiotensin receptor blockers, sacubitril/valsartan, beta blockers, digoxin, diuretics, and novel medications like sodium-glucose cotransporter 2 (SGLT2) inhibitors and mineralocorticoid receptor antagonists (MRAs), with the latter two being the focus of this review [3].

SGLT2 inhibitors, like dapagliflozin, empagliflozin, and sotagliflozin, are drugs used primarily to treat type 2 diabetes mellitus by inhibiting the sodium-glucose cotransporter in the proximal convoluted tubules, causing glycosuria, and leading to reduced blood glucose levels [7]. SGLT2 inhibition also leads to natriuresis, causing osmotic diuresis, which reduces the fluid overload and plasma volume in patients with heart failure [8]. In the context of heart failure, SGLT2 inhibition also reduces cardiac fibrosis, causes vasodilation, and reduces inflammation [9]. Multiple trials and meta-analyses have shown that SGLT2 inhibitors are beneficial in preventing and treating heart failure [10].

MRAs are of two types: steroidal (spironolactone, eplerenone, etc.) and non-steroidal (finerenone). MRAs inhibit the binding of aldosterone to mineralocorticoid receptors in the distal convoluted tubules and collecting ducts, causing increased natriuresis and potassium reabsorption, which in turn causes osmotic diuresis and a reduction in plasma volume [11]. MRAs also provide benefits in combating cardiac fibrosis and remodelling [12]. However, since they increase potassium reabsorption, MRAs are prescribed cautiously due to the risk of hyperkalemia [3]. Evidence is scarce on the long-term benefits of MRAs, and a knowledge gap exists when comparing the benefits of MRAs to those of SGLT2 inhibitors. Hence, this systematic review was undertaken to investigate and compare the long-term benefits of SGLT2 inhibitors versus MRAs in treating heart failure, regardless of ejection fraction and aetiology, by comparing the rates of worsening heart failure events and cardiovascular deaths.

## Review

Methods

This systematic review was conducted to compare the relative long-term benefits of SGLT2 inhibitors and MRAs. The review was adherent to the Preferred Reporting Items for Systematic Reviews and Meta-Analyses (PRISMA) guidelines [13] and registered on the International Prospective Register of Systematic Reviews (PROSPERO) (CRD420251137187). A comprehensive literature search was conducted on PubMed, MEDLINE, Scopus, and ClinicalTrials.gov for randomized controlled trials (RCTs) that evaluated the clinical effectiveness of SGLT2 inhibitors or MRAs when used to treat heart failure, published up to June 6, 2025. The search terms used were as follows: "empagliflozin", "dapagliflozin", "canagliflozin", "ertugliflozin", "sotagliflozin", "sodium-glucose transporter 2 inhibitors", "spironolactone", "eplerenone", "finerenone", "mineralocorticoid receptor antagonists", "heart failure", and "cardiovascular diseases". The detailed reproducible search strategies and filters used in the databases explored are illustrated in Table 1.

**Table 1:** Search strategies used in this review

Databases	Search strategies	Filters
PubMed	("empagliflozin" [Supplementary Concept] OR "dapagliflozin" [Supplementary Concept] OR "Canagliflozin/therapeutic use"[Mesh] OR "ertugliflozin" [Supplementary Concept] OR "Sodium-Glucose Transporter 2 Inhibitors/therapeutic use"[Mesh]) OR ("Spironolactone/therapeutic use"[Mesh] OR "Eplerenone/therapeutic use"[Mesh] OR "finerenone" [Supplementary Concept] OR "Mineralocorticoid Receptor Antagonists/therapeutic use"[Mesh]) AND ("Heart Failure"[Mesh] OR "Cardiovascular Diseases"[Mesh])	Published in the last 5 years, articles in English, randomized controlled trials
ClinicalTrials.gov	Condition/disease: Heart Failure; Intervention/treatment: Sodium-Glucose Transporter 2 Inhibitors OR Empagliflozin OR Dapagliflozin OR Ertugliflozin OR Canagliflozin OR Mineralocorticoid Receptor Antagonist OR Eplerenone OR Finerenone OR Spironolactone; Study status: All Studies	Study status: completed studies. Study type: interventional. Study results: with results. Results first posted: 06/06/2020 to 06/06/2025
Scopus	(("empagliflozin" OR "dapagliflozin" OR "canagliflozin” OR "ertugliflozin” OR "Sodium-Glucose Transporter 2 Inhibitors”) OR ("spironolactone” OR "eplerenone” OR "finerenone” OR "Mineralocorticoid Receptor Antagonists”)) AND ("Heart Failure" OR "Cardiovascular Diseases”) AND (“randomized controlled trials” OR “randomised controlled trials”)	Published in the last 5 years. Document type: articles. Language: English. Source type: journals

**Figure 1:**
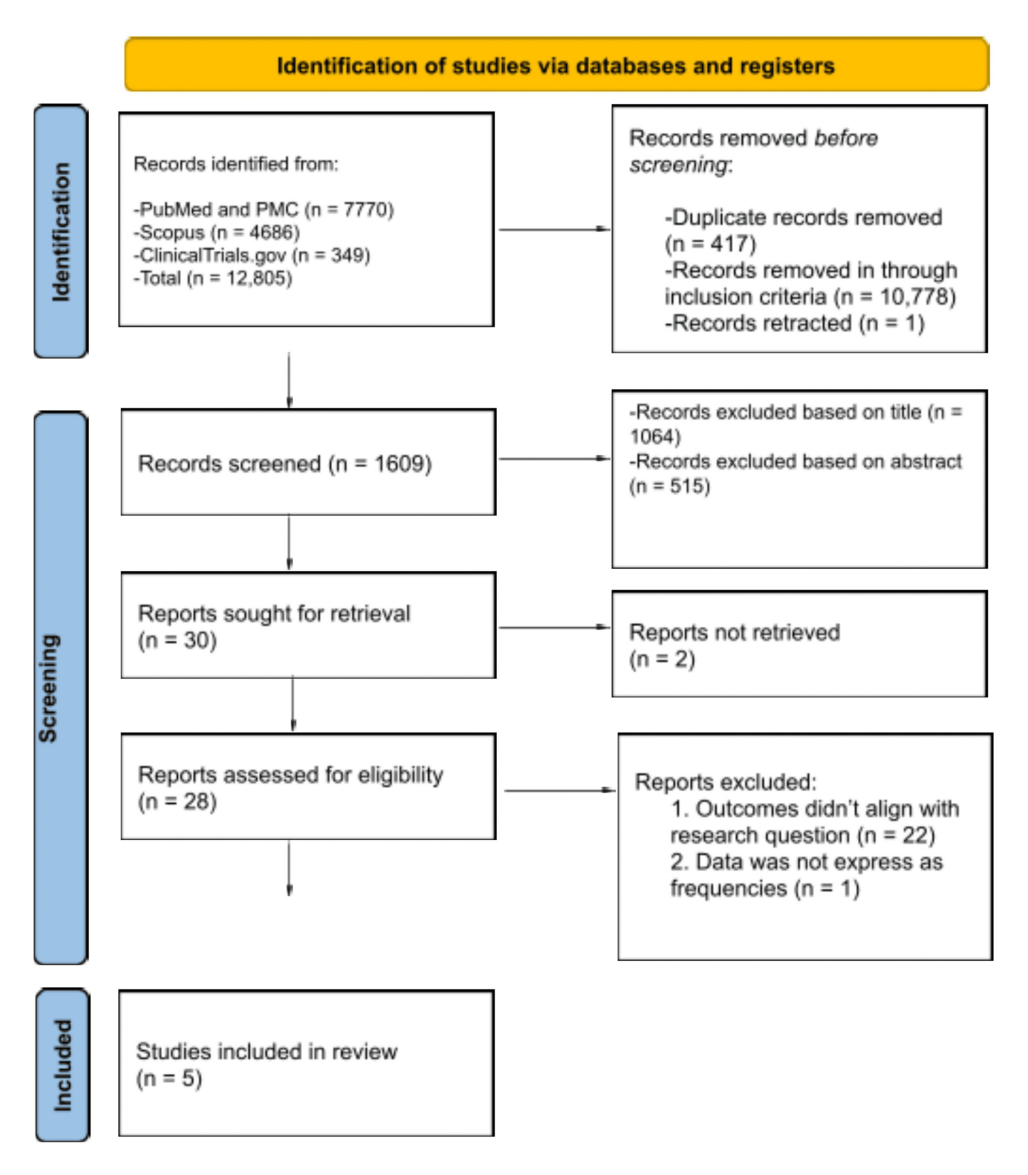
PRISMA flowchart PRISMA: Preferred Reporting Items for Systematic Reviews and Meta-Analyses

The inclusion criteria were RCTs, studies conducted in the last five years (since 2020), studies in English, studies on adults being treated for heart failure from any cause and any phenotype, and studies providing data on worsening heart failure events and cardiovascular deaths. The exclusion criteria were studies not published in English, studies published before June 2020, studies on <18-year-olds, studies whose population had patients at risk of heart failure, studies that didn't evaluate worsening heart failure events and cardiovascular deaths as outcomes, and studies that were grey literature, conference abstracts, and pre-prints. Using the citation manager Zotero (Corporation for Digital Scholarship, Vienna, Virginia, United States) and Microsoft Excel (Microsoft Corporation, Redmond, Washington, United States), two investigators independently reviewed the articles extracted from the databases. We excluded duplicates and screened titles, abstracts, and full texts and performed backward snowballing of reference lists for relevant articles. All disagreements were resolved through discussions with a third reviewer. The PRISMA flowchart in Figure 1 summarizes the search and study selection. Two investigators independently extracted data about the study, population characteristics, comparators, and outcomes. All disagreements were resolved through discussion and with input from the other investigators. One investigator assessed the risk of bias using the RoB 2 tool [14], and the findings were scrutinized by another reviewer. All the trials selected for the review had a low risk of bias (refer to Table 2 for a full breakdown of the quality assessment).

**Table 2:** Results of the quality assessment conducted using the RoB 2 tool

Trials	Random sequence generation	Allocation concealment	Blinding of participants and professionals	Blinding of outcome assessment	Incomplete outcome data	Selective reporting	Other biases
DELIVER [15]	Low	Low	Low	Low	Low	Low	Low
PRESERVED-HF [16] (exploratory clinical outcome)	Low	Low	Low	Low	Low	Low	Low
EMPULSE [17]	Low	Low	Low	Low	Low	Low	Low
SOLOIST-WHF [18]	Low	Low	Low	Low	Low	Low	Unsure
FINEARTS-HF [19]	Low	Low	Low	Low	Low	Low	Low

Results

The RCTs that were deemed relevant to the review were DELIVER [15], PRESERVED-HF [16], EMPULSE [17], and SOLOIST-WHF [18] for SGLT2 inhibitors and FINEARTS-HF [19] for MRAs. The outcomes this review assessed were worsening heart failure events, defined as the frequency of either an unplanned hospitalization for heart failure or an urgent visit for heart failure, and cardiovascular deaths that were adjudicated. All the trials included except PRESERVED-HF had their primary outcome related to worsening of heart failure, through win ratios, composite outcomes, and frequencies. PRESERVED-HF focused on changes in the Kansas City Cardiomyopathy Questionnaire-Clinical Summary Score (KCCQ-CSS) after the initiation of therapy, with worsening heart failure events assessed as an exploratory clinical outcome. All the trials included in the review assessed the frequency of cardiovascular deaths as part of a composite outcome, whereas in the case of PRESERVED-HF, all-cause mortality was assessed instead. The RoB 2 tool from Cochrane was utilized to conduct quality assessments, and all the trials were found to have a low risk of bias (refer to Table 2 for a full breakdown). In PRESERVED-HF, the risk of bias was assessed for the exploratory clinical outcome exclusively. In SOLOIST-WHF, the trial was stopped early, and cardiovascular deaths were investigator-reported instead of adjudicated, which could contribute to some bias.

This review studies a total population of 14340 patients, in which 4166 patients over four trials were in the SGLT2 inhibitor (3293 took dapagliflozin, 265 took empagliflozin, and 608 took sotagliflozin) treatment arms (4173 in the placebo treatment arm) and 3003 patients in one trial were in the MRA (finerenone) treatment arm (2998 in the placebo treatment arm). Baseline characteristics of populations included are in Table 3. Patients with heart failure, irrespective of phenotype and cause, were included in the study. While comparing the efficacies, to calculate the event rate of worsening heart failure events in the trials using SGLT2 inhibitors, a weighted average of events in both treatment arms was calculated. Using event rates, risk reduction and relative risk reduction were calculated for the population in SGLT2 inhibitor trials and FINEARTS-HF. From this information, we were able to calculate that SGLT2 inhibitors reduced the occurrence of worsening heart failure events by 25.21% compared to placebo and MRAs reduced it by 17.93%. Similar methods were used, and the reduction of the occurrence of cardiovascular death was assessed. SGLT2 inhibitors reduced the occurrence of cardiovascular deaths by 14.36% compared to placebo, and MRAs reduced it by 7.16%. The calculations for worsening heart failure events are illustrated in Tables 4-5, and the calculations for cardiovascular deaths are illustrated in Tables 6-7. 

**Table 3:** Details and baseline characteristics of the trials included in this review SGLT2: sodium-glucose cotransporter 2; MRAs: mineralocorticoid receptor antagonists; RCT: randomized controlled trial; KCCQ-TSS: Kansas City Cardiomyopathy Questionnaire-Total Symptom Score; eGFR: estimated glomerular filtration rate; KCCQ-CSS: Kansas City Cardiomyopathy Questionnaire-Clinical Summary Score; 6MWD: six-minute walking distance; HFmrEF: heart failure with mildly reduced ejection fraction; HFpEF: heart failure with preserved ejection fraction; NYHA: New York Heart Association; LVF: left ventricular function; T2DM: type 2 diabetes mellitus

Characteristics	SGLT2 inhibitors								MRAs	
Study	DELIVER [15]		PRESERVED-HF [16]		EMPULSE [17]		SOLOIST-WHF [18]		FINEARTS-HF [19]	
Author	Solomon et al.		Nassif et al.		Voors et al.		Bhatt et al.		Solomon et al.	
Type of study	RCT		RCT		RCT		RCT		RCT	
Publication year	2022		2021		2022		2021		2023	
Outcomes	Composite outcome of worsening heart failure and cardiovascular deaths, total heart failure-related hospitalizations, cardiovascular deaths, changes in KCCQ-TSS, change in eGFR slope		Changes in KCCQ-CSS, KCCQ-TSS, and 6MWD in 12 weeks. Exploratory clinical outcome: total worsening of heart failure events		Hierarchical composite of all-cause death, heart failure events, and KCCQ-TSS changes at 90 days. Components studied individually: worsening of heart failure events, cardiovascular deaths		Composite of worsening heart failure events and cardiovascular deaths, worsening heart failure events, cardiovascular deaths, all-cause deaths		Composite of cardiovascular deaths and worsening heart failure events, worsening heart failure events, cardiovascular deaths, all-cause mortality, change in KCCQ-TSS	
Study design	Randomly assigned 6263 patients with HFmrEF or HFpEF to receive either 10 mg of dapagliflozin or placebo		Randomly assigned 324 patients with HFpEF to receive either 10 mg of Dapagliflozin or placebo		Randomly assigned 530 patients who were admitted for acute heart failure or decompensated chronic heart failure to receive either 10 mg of empagliflozin or placebo		Randomly assigned 1222 patients who were admitted for decompensated heart failure to receive either 200 mg of sotagliflozin or placebo		Randomly assigned 6001 patients with HFmrEF or HFpEF to receive 20 mg/40 mg of finerenone or placebo	
Treatment arms	Dapagliflozin	Placebo	Dapagliflozin	Placebo	Empagliflozin	Placebo	Sotagliflozin	Placebo	Finerenone	Placebo
Number of patients	3131	3132	162	162	265	265	608	614	3003	2998
Baseline characteristics										
Median age	71.8±9.6	71.5±9.5	69	71	71	70	69	70	71.9	72
Males	1767	1749	70	70	179	172	410	400	1648	1621
Females	1364	1383	92	92	86	93	198	214	1355	1377
NYHA class I	NA	NA	NA	NA	8	6	NA	NA	NA	NA
NYHA class II	2314	2399	96	90	95	91	NA	NA	2081	2065
NYHA class III	807	724	65(III+IV)	72 (III+IV)	134	145	NA	NA	903	910
NYHA class IV	10	8	NA	NA	26	23	NA	NA	18	23
Median LVF %	54±8.6	54.3±8.9	60	60	31	32	35	35	52.6±7.8	52.5±7.8
Median NTpro-BNP	NA	NA	641	710	3299	3106	1816	1741	1053	1028
Estimated eGFR	61.9±19	61.9±19	56	54	50	54	49.2	50.5	61.9±19.4	62.3±20
Patients with T2DM	1401	1405	90	91	124	116	NA	NA	1217	1222
Worsening heart failure events (event rate)	368 (0.1175)	455 (0.1452)	9 (0.0555)	9 (0.0555)	36 (0.1358)	52 (0.1962)	194 (0.3190)	297 (0.4837)	842 (0.2803)	1024 (0.3415)
Cardiovascular deaths (event rate)	231 (0.0737)	261 (0.0833)	NA	NA	11 (0.0415)	22 (0.08301)	28 (0.0460)	33 (0.0537)	242 (0.0805)	260 (0.0867)

**Table 4:** Calculations for worsening heart failure in trials studying SGLT2 inhibitors SGLT2: sodium-glucose cotransporter 2

SGLT2 inhibitors	
Weighted average of the event rate in the SGLT2 arm	0.1457
Weighted average of the event rate in the placebo arm	0.1948
Relative risk	0.7479
Relative risk reduction	0.2521
% reduced compared to placebo	25.21%

**Table 5:** Calculations for worsening heart failure events in trials studying MRAs MRAs: mineralocorticoid receptor antagonists

MRAs	
Event rate in the MRA arm	0.2803
Event rate in the placebo arm	0.3415
Relative risk	0.8207
Relative risk reduction	0.1793
% reduced compared to placebo	17.93%

**Table 6:** Calculations for cardiovascular deaths in trials studying SGLT2 inhibitors SGLT2: sodium-glucose cotransporter 2

SGLT2 inhibitors	
Weighted average of the event rate in the SGLT2 arm	0.0674
Weighted average of the event rate in the placebo arm	0.0787
Relative risk	0.8564
Relative risk reduction	0.1436
% reduced compared to placebo	14.36%

**Table 7:** Calculations for cardiovascular deaths in trials studying MRAs MRAs: mineralocorticoid receptor antagonists

MRAs	
Event rate in the MRA arm	0.0805
Event rate in the placebo arm	0.0867
Relative risk	0.9284
Relative risk reduction	0.0716
% reduced compared to placebo	7.16%

Discussion

In this systematic review, which included 14340 patients over five trials, we were able to conclude that SGLT2 inhibitors and MRAs reduced the risk of worsening heart failure events and cardiovascular deaths compared to placebo [15-19]. When comparing the two classes of drugs, SGLT2 inhibitors show a greater reduction of risk than MRAs. This systematic review does come with its limitations. Firstly, we were very rigid when it came to including studies that assessed worsening of heart failure events; this led us to exclude studies that only assessed first-time re-hospitalization rates and did not have data about recurrent admissions and urgent care visits. The earlier limitation led us to include data from five trials, four trials studying SGLT2 inhibitors (8339 patients including placebo) and only one trial studying MRAs (6001 patients including placebo). Three SGLT2 inhibitors were assessed (dapagliflozin, empagliflozin, and sotagliflozin), while only one MRA was assessed (finerenone). It should also be noted that EMPULSE and SOLOIST-WHF enrolled patients with heart failure with reduced ejection fraction (HFrEF) with a median left ventricular ejection fraction (LVEF) of around 31% and 35%, respectively, while DELIVER, PRESERVED-HF, and FINEARTS-HF enrolled patients with heart failure with preserved ejection fraction (HFpEF) or heart failure with mildly reduced ejection fraction (HFmrEF) [15-19]. We were also limited to articles that were published in English and didn't include articles published in other languages.

Prior research has evaluated and contrasted the long-term efficacy of individual SGLT2 inhibitors and SGLT2 inhibitors versus angiotensin receptor-neprilysin inhibitors in patients with heart failure [20]. This review has, for the first time to our knowledge, compared the efficacies of SGLT2 inhibitors and MRAs using RCTs, hence filling the knowledge gap that exists in the long-term benefits of initiating SGLT2 inhibitors and MRAs in heart failure and a head-to-head comparison between the two classes of drugs. The results from this review can be used to guide clinicians who want to initiate these medications to treat heart failure and reinforce the knowledge that already exists about this topic. We believe that further research is needed on this topic, particularly through comparative analyses of outcomes like median change in LVEF, NT-proBNP levels, estimated glomerular filtration rate (eGFR), Kansas City Cardiomyopathy Questionnaire-Total Symptom Score (KCCQ-TSS), KCCQ-CSS, and six-minute walking distance (6MWD). A meta-analysis of the outcomes assessed in this review and the other outcomes mentioned previously would be needed to give us the best tools to comprehensively compare the efficacies of the two drug classes. Another gap in research that was identified was the lack of meta-analyses comparing the efficacies of individual MRAs.

## Conclusions

Both SGLT2 inhibitors and MRAs significantly reduce the risk of worsening heart failure and cardiovascular death when compared with placebo. Across available randomized trials that compared effectiveness indirectly, SGLT2 inhibitors appear to confer greater relative benefit in long-term outcomes. Direct comparative studies are required to confirm these findings.
